# Effects of BFR-RST on upper limb performance in boxers: a study based on physiological indices, anthropometric measurement indices, anaerobic power, and punching performance

**DOI:** 10.3389/fphys.2025.1453153

**Published:** 2025-05-13

**Authors:** QingLou Xu, Jiaming Fei, Ruiqiu Mao, Lei Liu, Cheng Fei

**Affiliations:** ^1^ Department of Physical Education, Zhejiang Guangsha Vocational and Technical University of Construction, Jinhua, Zhejiang, China; ^2^ School of Physical Education, Huainan Normal University, Huainan, Anhui, China; ^3^ Graduate Department, Shenyang Sport University, Shenyang, Liaoning, China; ^4^ Department of Cultural Management, Moscow State University, Moscow, Russia

**Keywords:** combat sports, blood flow restriction, repeated-sprint training, wingate test, peak punching speed

## Abstract

**Introduction:**

This study examined the effects of a four-week, 40% arterial occlusion pressure blood flow restriction-specific repeated sprint training (RST) regimen on the upper-limb anaerobic capacity and punching performance of male collegiate boxers.

**Methods:**

Thirty-six healthy participants were assigned to either a blood flow restriction training group (Experimental Group, EG, n = 18) or a conventional training group (Control Group, CG, n = 18). Physiological measurements indices and anthropometric measurement indices (resting heart rate, heart rate after Wingate test, upper arm tensed circumference, upper arm relaxed circumference, BPP), upper-limb anaerobic power indices (PP, MP, tPP, PD), and punching performance indices (peak punching speed, total number of 6s all-out punches, peak punching speed post 6s all-out punches) were recorded at T0 (pre-intervention), T1 (mid-intervention), and T2 (post-intervention).

**Results:**

Two-way repeated measures ANOVA revealed that, compared to the CG, the EG showed significant increases at T2 in upper arm tensed circumference (+4.6%), BPP (+11.24% at T1, +10.18% at T2), PP (+10.09% at T1, +9.03% at T2), MP (+12.29% at T1, +11.69% at T2), tPP (+16.00% at T1, +8.09% at T2), total number of 6s all-out punches (+8.4% at T2), and peak punching speed post 6s all-out punches (+10.7% at T2). In contrast, heart rate after the Wingate test (−3.2% at T2) and PD (−6.57% at T1, −5.59% at T2) decreased (p < 0.05), with significant group-by-time interactions observed at T2 (p < 0.05).

**Discussion:**

This study demonstrates that both types of RST training effectively enhance upper-limb anaerobic power and strength in boxers, with the improvements from blood flow restriction training proving superior to those from conventional RST. Furthermore, while neither training method significantly affected peak punch speed, the BFR-RST program significantly outperformed conventional RST in terms of fatigue resistance, leading to notable enhancements in anaerobic power.

## 1 Introduction

Boxing, an intermittent sport, demands both aerobic and anaerobic capacities. Athletes engage in high-intensity activities like repetitive punching, footwork, dodging, or defending, typically lasting no more than 10 s and utilizing almost all muscle groups. Recovery within the brief rest periods, often under 30 s, is typically incomplete. The ability to sustain performance across multiple sprints in a match, termed repeated-sprint ability (RSA), is crucial. RSA’s physiological limitations include diminished force production, inadequate energy supply, and the accumulation of metabolites (Mckee et al., 2023). Therefore, effective RSA training should focus on enhancing both aerobic and anaerobic metabolic capacities. This approach optimizes ATP supply during frequent sprint repetitions.

Given these physiological aspects, repeated sprint training (RST) serves as a crucial method to foster physiological adaptation in boxers. This training typically includes short “all-out” efforts (<10 s) with incomplete recoveries (<60 s or a work-to-rest ratio <1:4) (Girard et al., 2011). Sprint high-intensity interval training, in contrast to traditional high-intensity interval training, can induce significant physiological adaptations within weeks, potentially improving power, speed, repeated-sprint ability, and endurance (Taylor et al., 2015). However, most RST studies focus on the impact of lower-limb activities in various ball and team sports (Eniseler et al., 2017; Rampinini et al., 2009; Aziz et al., 2008), with limited research on upper-limb RST. Boxing relies heavily on upper-limb muscles. Although the muscle mass in the upper limbs is smaller, studies comparing similarly trained upper and lower limb muscles have found that the muscle fibers in the arms exhibit higher specific force capabilities compared to those in the legs (Gejl et al., 2021). Therefore, applying blood flow restriction (BFR) training to the upper limbs may lead to unique physiological responses in boxing. For example, peak oxygen uptake in upper limb exercises is lower, with slower oxygen uptake kinetics, possibly due to structural differences such as fewer capillaries in the upper limbs (Zinner et al., 2016). Therefore, upper limb RST with blood flow restriction (BFR) might offer enhanced benefits by targeting these physiological factors. However, the mechanisms underlying the effects of BFR-RST on the upper limbs remain unclear, with only a few studies addressing BFR-RST, most of which have focused solely on the acute responses in athletes. One study observed a decrease in repeated-sprint ability in the upper limbs following short-term BFR-RST (Peyrard et al., 2019), while another report indicated that short-term BFR-RST led to changes in deoxygenated hemoglobin concentration and total hemoglobin content in the arms, enhancing vascular regulation, with arms being more sensitive to reduced oxygen supply from hypoxia than the legs (Willis et al., 2019). Overall, current literature suggests that BFR-RST research in upper-limb exercises is limited, and there are significant differences in the effects between the lower and upper limbs. To date, no studies have applied BFR-RST to the upper limbs of boxers. We aim to validate its effects on anaerobic power and punching performance in the upper limbs over a 4-week training period, forming the basis of this research.

The primary objective of this study is to provide reliable references for the daily training of coaches and athletes and to generate research insights for future studies on BFR. We hypothesize that: 1) a 4-week BFR-RST regimen will significantly enhance the anaerobic power of boxers’ upper limbs; 2) these improvements in anaerobic power will positively influence the punching performance of boxers under fatigue conditions.

## 2 Materials and methods

### 2.1 Experimental approach to the problem

During the 4-week intervention, training sessions were conducted three times a week (Tuesday, Thursday, and Sunday). The training protocols for both the EG and CG were identical except for the application of BFR. The modified Borg CR-10 Subjective Fatigue Scale (RPE 0–10) was employed to maintain consistent training intensity across both groups (Tibana et al., 2018). Coaches and athletes received training on this scale before the intervention commenced.

Each training session followed a standardized design, based on protocols from the existing literature (Kamandulis et al., 2018). Initially, athletes completed 15 min of warm-up exercises and dynamic stretching. This was followed by 15 min of moderate-intensity (RPE-5) punching practice without target contact, and 25 min of combat technique sparring (RPE-7) focusing on technical enhancement under an experienced trainer’s supervision. Subsequently, the EG and CG participated in separate 15-min sessions of technical sandbag training. The EG underwent three rounds of 14 sets, each lasting 3 s (with a 10-s rest between sets, passive recovery, and a 1-min rest between rounds) of high-intensity (RPE-10) specialized repeated sprint training (hitting the sandbag) with blood flow restriction. In contrast, the CG performed the same exercises without blood flow restriction. All athletes concluded the session with 10 min of relaxed shadowboxing and static stretching. Before the intervention, at 2 weeks, and at 4 weeks, physiological indicators, upper limb anaerobic power, and punching performance tests were simultaneously administered to both groups.

During the experiment, participants were instructed to (1) ensure a minimum of 8 hours of sleep before each test; (2) maintain their usual dietary and hydration habits in the days leading up to the test; (3) avoid beverages containing caffeine or other stimulants; and (4) refrain from any training outside the intervention training.

### 2.2 Subjects

According to the G*power sample size estimation software, setting Power = 0.8, α = 0.05, and two groups, the total sample size was estimated to be 34, with 17 participants in each group. After recruitment and screening, a total of 36 male athletes from Shenyang Sport University, specializing in martial arts and boxing, agreed to participate in the study. These athletes had competed in at least ten official amateur or college boxing matches and had been training in boxing since high school or earlier. Before the trials, they completed a questionnaire about their boxing experience and recent training activities. Measurements such as height, weight, and overall health were recorded to exclude any injuries that might affect the test results. A medical examination, adhering to protocols from prior research, was conducted to verify their eligibility for the blood flow restriction experiment and confirm robust cardiovascular health (Patterson et al., 2019). Subsequently, participants were evenly divided into the experimental group (EG, n = 18) and the control group (CG, n = 18). An independent sample t-test confirmed that there were no statistical differences in age, height, weight, and other baseline characteristics between the groups (Table 1). This experiment was conducted in compliance with the Declaration of Helsinki, written informed consent was obtained from all participants, and the Ethics Committee of Shenyang Sport University approved the study (Ethics [2024] No. 12).

**TABLE 1:** Grouping information of subjects (n = 18).

Variable	EG	CG	*t*	*P*	*d*
Age (years)	19.67 ± 0.4	20.11 ± 1.0	−1.733	0.097	0.594
Height (cm)	173.3 ± 6.3	174.5 ± 4.7	−0.648	0.522	0.222
Weight (kg)	68.3 ± 10.6	66.4 ± 8.9	0.582	0.564	0.200
Upper limb length (cm)	66.8 ± 2.9	65.2 ± 2.8	1.684	0.101	0.578
BMI(kg/m^2^)	22.2 ± 2.9	21.2 ± 2.2	1.166	0.252	0.400
Years of training (years)	6.57 ± 1.4	6.87 ± 1.2	−0.690	0.495	0.237

### 2.3 Procedures

#### 2.3.1 Upper-limb BFR

**FIGURE 1:**
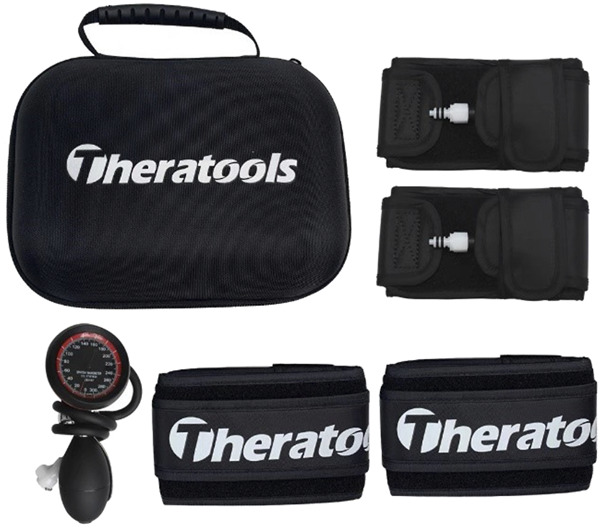
Blood pressure cuff.

A blood pressure cuff (Theratools, China, 68 cm length, 7.5 cm width) was placed on the upper portion of the biceps brachii for BFR (Figure 1). It is crucial to note that the pressure exerted by the cuff varied for each participant. It is generally recommended to maintain a pressure range of 80–180 mmHg when exercising the upper or lower limbs. A professional calculation method involves using Doppler ultrasound to monitor the pulse at the radial artery of the upper limb, which determines the required pressure (Mouser et al., 2017). The cuff was gradually inflated until the radial artery pulse was no longer detectable, establishing the limb occlusion pressure (LOP) for each subject (this step is completed during the physical examination of recruited participants). Typically, exercises for the upper limb use 40%–80% LOP(WEI et al., 2019). However, initial tests in this study indicated significant discomfort among athletes at 50% LOP during the RST phase, leading to a reduction to 40% LOP. Athletes experiencing discomfort generally adapted after two sessions, enabling them to complete the training regimen.

#### 2.3.2 Experimental measures

The research process encompasses three assessments: Pre-intervention, Mid-intervention (second week), and Post-intervention (fourth week). All testers are professionally trained and well-versed in the operation and usage of the equipment. Technical training is provided to participants in both the experimental group (EG) and control group (CG) before the tests. On the day of testing, the procedures are meticulously adhered to as outlined. To minimize testing errors, all three tests are administered by the same team of testers.

#### 2.3.3 Physiological index testing and anthropometric measure testing

The resting heart rate test is conducted between 7:00–8:00 a.m., in a fasted state, in a seated and awake position, using a heart rate monitor (EZON, Fujian, China). Following the resting heart rate test, the upper arm circumference is measured in both tensed and relaxed states using a tape measure. The measurement procedure is as follows: 1. The participant stands or sits with the arm naturally hanging down, relaxed. 2. The midpoint between the acromion (the highest point of the shoulder, located on the outer side of the scapula) and the olecranon (the most prominent bony point of the elbow, located at the proximal end of the ulna) is identified (usually at the most prominent point of the biceps). 3. A tape measure is wrapped around the arm horizontally, ensuring that it is snug against the skin but not too tight. 4. Measurements are taken both in the relaxed state (with the arm fully extended at 180°) and in the tensed state (with the arm bent at 90°), and the maximum circumference value is recorded.

Subsequently, the upper limb power measurement test is scheduled between 9:00 and 11:00 a.m. Before the test, all participants are instructed to warm up and observe a demonstration of the movements to ensure safety and proficiency. The warm-up procedure is as follows: 1. Participants, dressed in athletic wear, engage in a 10-min slow jog and stretching activities; 2. A 2–4 min warm-up on the machine with no resistance is performed, during which 2–3 maximal efforts are completed to ensure adequate preparation.

The bench press means propulsive power (BPP) is utilized to assess the upper limb power of participants (Sanchez-Medina et al., 2010). These tests are performed on a Smith machine to eliminate the influence of horizontal displacement on the results. The study employs a power assessment system based on velocity-based training (VBT), the GymAware device, to measure changes in the output power of the upper limb bench press before and after the intervention. GymAware, which attaches to the barbell or plate-loaded strength equipment, uses a linear position sensor to calculate displacement and time, thereby computing velocity and average velocity to determining peak power. The reliability of the GymAware system in monitoring training intensity has been confirmed in numerous studies (GRGIC et al., 2020). Participants are instructed to execute the movement at maximum speed three times under each load, starting at 30% of body weight and increasing the load by 5% in each set until a decline in BPP is noted (approximately two to five sets), with a 1-min rest between sets. During the test, participants control the lowering of the barbell until it lightly touches the chest, then explosively move the barbell as quickly as possible on command to achieve the highest average propulsive power. The highest BPP achieved during the test is included in the experimental data.

#### 2.3.4 Wingate testing

The Wingate test, a 30s all-out exercise under constant load, is a common method for assessing athletes’ anaerobic performance (Julio et al., 2019). Previous studies have systematically compared two different upper-limb Wingate anaerobic testing systems (Cranlea and Monark), showing a strong correlation across all parameters (r = 0.99–0.97; P < 0.001), confirming the relative reliability and reproducibility of the test (Talbot et al., 2014). This test was performed using a MONARK 891E upper-limb anaerobic power machine (Sweden), with a resistance load set at 5% of the participant’s body weight. Participants wear a heart rate monitor during the test. Initially, a 3-min warm-up is performed on the device. Once the heart rate reaches 150–160 bpm, participants begin to vigorously operate the power crank for 30 s. During this interval, peak power (PP), minimum power (MP), time to reach peak power (tPP), and the rate of anaerobic power decline (PD) are recorded. Test data are automatically collected and stored on a computer using the MONARK test software (Monark Anaerobic Test Software, Version 3.3.0.0). Following the test, participants undergo passive recovery in the same position, and heart rate is measured after 30 s. The test is scheduled for the evening of the same day, from 7:00 to 9:00 p.m. Prior to the test, subjects underwent comprehensive preparation and stretching and performed two to three preliminary loading exercises to familiarize themselves with the machine.

#### 2.3.5 Punching performance testing

A three-dimensional radiographic framework (013-c) and two high-speed cameras (Sony HVR-V1C, Japan, 200 Hz) were used to capture the dynamic punching movements of athletes in a fixed position. The cameras were placed directly in front of and to the right of the athlete. The two-dimensional calibration data captured by the cameras were processed using Direct Linear Transformation (DLT) to obtain kinematic parameters related to the athlete’s punching motion. The analysis was performed using the APAS motion analysis software (Ariel, United States), and the acquired data were smoothed using a low-pass filter (frequency: 10 Hz). This method has been shown to be reliable and reproducible (Qinglou et al., 2023).

The experimental procedures unfolded at the boxing training facility of Shenyang Sport University, prior to the commencement of the tests, participants underwent thorough warm-up sessions, and the experimental procedures were diligently demonstrated to ensure a clear understanding. The test is scheduled for the afternoon of the same day from 2:00 to 5:00 p.m. The first test conducted was the peak punching speed test, where athletes executed approximately three cross punches into the air from a stationary regular guard position, regulating the interval between each punch. The peak speed of these punches was recorded. This was followed by a punching frequency test, where subjects were required to deliver punches at maximum frequency (alternating jabs and crosses at a target) for 6 s upon command. The total number of punches thrown was tallied (Only crosses). Immediately after, a retest for peak punching speed was conducted to assess performance following upper-limb fatigue. Subjects were given a substantial rest period (>2 min) before each test. To determine the peak punching speed in the recorded video, the starting frame was selected as the first frame in which the participant initiated the punching motion, and the ending frame was the last frame when the hand no longer moved forward and reached the maximum punching distance.

### 2.4 Statistical analyses

Statistical analysis was performed using SPSS 25.0 (IBM, Corp, Armonk, NY, United States), and the results were reported as mean ± SD. The Shapiro-Wilk (SW) test was used for assessing data normality, while the Levene test evaluated the homogeneity of variances. During the experimental grouping, an independent samples t-test confirms the absence of significant differences between groups. A two-factor repeated measures ANOVA analyzes the main effects and interaction effects of group and time on the three test datasets. If the interaction effect is significant, a simple effects analysis is conducted. A p-value less than 0.05 is considered indicative of statistically significant differences. Post hoc analyses were performed using the Least Significant Difference (LSD) test, a straightforward variant of the t-test. Unlike other tests, LSD does not adjust for test level but uses the entire sample information for more accurate standard error estimation, with P < 0.05 indicating statistical significance.

## 3 Result

No adverse events, such as dizziness or injuries, were reported during the study. All athletes, including those who experienced significant discomfort in their upper limb muscles during the first two RST-BFR sessions, completed the prescribed training regimen. After 4 weeks of intervention training and three evaluations, Tables 2–4 summarize the differences between the two groups in upper limb physiological indicators and anthropometric measurement indicators, punching performance, and anaerobic power.

**TABLE 2:** Physiological indicators and anthropometric measurement indicators.

Variable	Group	T0	T1	T2
Resting heart rate (times/min)	EG	56.11 ± 1.71	56.00 ± 1.68	55.94 ± 1.55
	CG	54.94 ± 1.83	55.00 ± 1.50	55.22 ± 1.66
	F group/time/interaction 6.268/0.030/0.213			
	*P* group/time/interaction 0.017/0.970/0.809			
	*Partialη* ^*2*^ group/time/interaction 0.156/0.001/0.006			
Heart rate after wingate test (times/min)	EG	179.44 ± 7.01	176.28 ± 6.27	173.78 ± 5.87#
	CG	176.94 ± 6.19	176.67 ± 6.48	177.33 ± 6.62
	*F* group/time/interaction 0.124/1.772/2.264			
	*P* group/time/interaction 0.727/0.178/0.112			
	*Partialη* ^*2*^ group/time/interaction 0.004/0.050/0.062			
Upper arm tensed circumference (cm)	EG	29.01 ± 1.28	29.36 ± 1.21#	30.43 ± 1.31#∆
	CG	30.24 ± 2.59	30.26 ± 2.51	30.42 ± 2.58
	*F* group/time/interaction 1.110/89.437/52.938			
	*P* group/time/interaction 0.299/<0.001/<0.001			
	*Partialη* ^*2*^ group/time/interaction 0.032/0.725/0.609			
Upper arm relaxed circumference (cm)	EG	26.15 ± 1.55	26.16 ± 1.46	26.31 ± 1.36
	CG	27.17 ± 2.48	27.27 ± 2.43	27.31 ± 2.35
	*F* group/time/interaction 2.463/3.715/0.558			
	*P* group/time/interaction 0.126/0.039/0.575			
	*Partialη* ^*2*^ group/time/interaction 0.068/0.099/0.016			
BPP(W)	EG	354.89 ± 40.25	394.79 ± 41.58#	434.96 ± 36.29*#∆
	CG	361.78 ± 27.59	371.74 ± 27.31#	385.55 ± 28.30#∆
	*F* group/time/interaction 3.774/701.325/206.353			
	*P* group/time/interaction 0.060/<0.001/<0.001			
	*Partialη* ^*2*^ group/time/interaction 0.100/0.954/0.859			

Note: at pre-intervention (T0), mid-intervention (T1), and post-intervention (T2). Compared to the CG group, *P < 0.05. Within the group, compared to T0, #P < 0.05; compared to T1, ∆P < 0.05.

**TABLE 3:** Punching performance.

Variable	Group	T0	T1	T2
Peak punching speed (m/s)	EG	8.20 ± 0.66	8.23 ± 0.62	8.29 ± 0.53
	CG	8.30 ± 0.64	8.29 ± 0.35	8.29 ± 0.32
4	*F* group/time/interaction 0.098/0.262/0.404			
9	*P* group/time/interaction 0.757/0.770/0.669			
2	*P*artialη^2^ group/time/interaction 0.003/0.008/0.012			
Total number of 6s all-out punches (times)	EG	11.33 ± 0.91	11.50 ± 0.86	12.28 ± 0.75#
	CG	11.61 ± 1.14	11.78 ± 1.00	11.78 ± 0.94
7	*F* group/time/interaction 0.011/9.730/6.007			
8	*P* group/time/interaction 0.916/0.001/0.008			
0	*Partialη* ^*2*^ group/time/interaction <0.001/0.223/0.150			
Peak punching speed post 6s all-out punches (m/s)	EG	6.70 ± 0.56	7.22 ± 0.72*#	7.50 ± 0.42*#
	CG	6.56 ± 0.40	6.80 ± 0.34#	7.00 ± 0.36#
68	*F* group/time/interaction 6.601/35.251/3.168			
82	*P* group/time/interaction 0.015/<0.001/0.082			
85	*Partialη* ^*2*^ group/time/interaction 0.163/0.509/0.085			

Note: at pre-intervention (T0), mid-intervention (T1), and post-intervention (T2). Compared to the CG, group, *P < 0.05. Within the group, compared to T0, #P < 0.05; compared to T1, ∆P < 0.05.

**TABLE 4:** Anaerobic power.

Variable	Group	T0	T1	T2
PP(W)	EG	446.68 ± 39.34	491.76 ± 46.99*#	536.15 ± 38.70*#∆
	CG	443.33 ± 26.70	456.77 ± 35.58#	468.86 ± 39.33#∆
	*F* group/time/interaction 7.926/357.935/110.622			
	*P* group/time/interaction 0.008/<0.001/<0.001			
	*Partialη* ^*2*^ group/time/interaction 0.189/0.913/0.765			
MP(W)	EG	246.39 ± 48.22	276.69 ± 39.03#	309.02 ± 38.60*#∆
	CG	251.02 ± 31.23	261.71 ± 28.34#	272.62 ± 26.34#∆
	*F* group/time/interaction 1.754/190.108/45.132			
	*P* group/time/interaction 0.194/<0.001/<0.001			
	*Partialη* ^*2*^ group/time/interaction 0.049/0.848/0.570			
tPP(s)	EG	6.50 ± 2.45	7.54 ± 2.43#	8.15 ± 2.31*#∆
	CG	6.36 ± 2.34	6.37 ± 2.30	6.39 ± 2.30
	*F* group/time/interaction 1.735/34.279/32.065			
	*P* group/time/interaction 0.197/<0.001/<0.001			
	*Partialη* ^*2*^ group/time/interaction 0.049/0.502/0.485			
PD (%)	EG	46.77 ± 6.76	43.69 ± 3.94#	41.25 ± 3.74*#∆
	CG	46.06 ± 6.39	45.71 ± 6.74	45.50 ± 6.44
	*F* group/time/interaction 0.827/22.488/10.484			
	*P* group/time/interaction 0.369/<0.001/<0.001			
	*Partialη* ^*2*^ group/time/interaction 0.024/0.398/0.236			

Note: pre-intervention (T0), mid-intervention (T1), post-intervention (T2). Compared to the CG, group, *P < 0.05. Within the group, compared to T0, #P < 0.05; compared to T1, ∆P < 0.05.

The test results for physiological indicators showed no differences in resting heart rate or relaxed upper arm circumference before and after training for both groups. This aligns with our expectations, as resting heart rate primarily reflects an individual’s basal metabolic state and cardiorespiratory function, with significant changes typically requiring longer durations (e.g., several months) of aerobic training or high-intensity interval training (HIIT) interventions (Buchheit and Laursen, 2013). Therefore, the 4-week training intervention may not have been long enough to induce significant changes in resting heart rate.

No within-group differences were observed in heart rate after Wingate test or tensed upper arm circumference in the control group at T1 and T2, whereas the experimental group showed within-group differences at T2, with changes of −3.2% and 4.6%, respectively, compared to T0. Both groups showed significant improvements in BPP at T1 and T2, with the EG increasing by 11.24% and 10.18%, and the CG by 2.75% and 3.71%, respectively. The EG also showed significant differences compared to the CG at T2. We speculate that the greater physiological adaptations observed in the EG, such as muscle hypertrophy and increased recruitment of type II (fast-twitch) muscle fibers, were likely due to the application of blood flow restriction (BFR). The changes in resting heart rate and relaxed upper arm circumference were statistically significant (p < 0.05), and both time and group-time interactions had significant effects on tensed upper arm circumference and BPP (p < 0.05). These results suggest that the improvements in BPP, which reflect enhanced upper-limb explosive power, are likely due to BFR-induced physiological adaptations. This improvement in BPP is crucial for boxers’ punching speed and power, providing a foundation for maintaining high punching efficiency under fatigue.

In the Wingate test, both groups showed significant improvements in PP and MP at T1 and T2 (p < 0.05), with the EG’s increases exceeding 10% and the CG’s below 5%. The EG also showed significant differences compared to the CG at T1 and T2 (p < 0.05). The CG showed no changes in tPP and PD, while the EG showed both intra- and intergroup differences at T1 and T2 (p < 0.05). Furthermore, the main effect of training group on PP was significant (p < 0.05), and both time and group-time interactions had significant effects on PP, MP, tPP, and PD (p < 0.05).

In the punching performance test, no changes in peak punch speed were observed for either group at any of the three time points. The CG showed no changes in the total number of punches over 6 s, while the EG showed an 8.4% increase at T2 compared to T0, though no differences were observed between groups. Both groups exhibited increases in peak punch speed after 6 s at T2 compared to T0, with increases of 10.7% for the EG and 6.3% for the CG. Intergroup differences were found at T1 and T2. The main effect of training group on peak speed after 6 s was significant (p < 0.05), as were the effects of time and the group-time interaction on the total number of punches and peak speed after 6 s (p < 0.05).

Table 2: The experimental group showed significant changes in heart rate after Wingate test, Upper arm tensed circumference, and BPP, while the control group changed less. There were no significant differences in resting heart rate and Upper arm relaxed circumference between the two groups.

Table 3: The experimental group showed significant improvements in total number of 6s all-out punches and peak punching speed post 6s all-out punches, while the control group showed little change. There was no significant difference in peak punching speed between the two groups.

Table 4: The experimental group showed significant improvements in peak power (PP), minimum power (MP), time to reach peak power (tPP), and the rate of anaerobic power decline (PD) after the intervention (T2), while the control group showed little change.

## 4 Discussion

This study compared the effects of a 4-week repeated sprint training (RST) program under blood flow restriction state. Despite the RST’s low work-rest ratio (approximately 1:3, i.e., 14 sets of 3-s all-out punching with 10 s rest between sets) and the absence of a resistance load, both EG and CG reached peak fatigue levels (RPE = 10) during the intervention. Although these fatigue levels slightly differ from those in real-world boxing matches, it was noted that the total number of punches in elite men’s amateur boxing matches is significantly lower than in simulated training (Smith, 2006). Anaerobic capacity is highly correlated with competitive performance in boxing (Guohuan et al., 2022), suggesting that increased fatigue resistance—the ability to resist both physiological and psychological fatigue during prolonged or high-intensity exercise—could be advantageous in actual matches. This capacity directly affects athletes’ performance, recovery efficiency, and long-term training adaptation.

### 4.1 Physiological indicators and anthropometric measurement indicators

Based on the results of physiological indicators and anthropometric measurements, we found a clear contrast with the study by Mouser et al., who reported that combining blood flow restriction with high-intensity strength training did not enhance muscle strength compared to high-intensity strength training alone (Mouser et al., 2017). This discrepancy could be attributed to differences in training intensity and duration. Specifically, our study excluded resistance load, whereas Mouser et al., study involved a substantial resistance load (80% of 1RM), likely causing high muscular tension and consequent blood flow restriction. Yamada et al. found that loads exceeding 40%–50% of maximal voluntary contraction (MVC) can produce pressures exceeding systolic pressure, thereby obstructing blood flow (Kamandulis et al., 2018). Thus, the occlusion caused by high-intensity resistance training may interfere with the effects of blood flow restriction, leading to variations in study outcomes. However, other studies align with our findings. Abe et al. reported that a 3-week BFR walking training (14 min, 160–230 mmHg cuff pressure) increased the quadriceps cross-sectional area (+6%), maximum repeated leg press repetitions (+7%), and maximum isometric knee extension strength (+10%) (Abe et al., 2008). Additionally, 6 weeks of high-intensity BFR running increased the thickness of the rectus femoris (+8%) and the leg strength development compression rate (+25%), while unrestricted training showed minimal changes (0% and +2%, respectively) (Behringer et al., 2017). Our findings are consistent with these results, showing that BFR-RST is superior to traditional RST in inducing greater adaptive responses under the same training conditions.

Although some studies suggest that improvements in muscle strength following BFR training may stem from increases in muscle cross-sectional area (Scott et al., 2014), there remains some controversy regarding the causal relationship between muscle hypertrophy and strength gains (Reggiani and Schiaffino, 2020). Recent systematic reviews and meta-analyses have shown that BFR resistance exercise (BFR-RE) effectively enhances skeletal muscle strength and/or hypertrophy (cross-sectional area, CSA) in both healthy young (Martin-Hernandez et al., 2013; Ozaki et al., 2013) and older populations (Libardi et al., 2015; Vechin et al., 2015; Lixandrão et al., 2017). Although the increase in CSA might initially be attributed to acute edema during and shortly after BFR (<1 week), most studies indicate sustained improvements following more than 3 weeks of systematic BFR training (Fujita et al., 2008; Jakob et al., 2012). For boxers, the practical significance of increasing arm circumference means that their strength or explosive force has changed. In addition, the increase in arm circumference may enhance the self-confidence and competitive state of athletes, directly improving their performance in the competition. Takarada et al. found that blood flow restriction at 100 mmHg pressure increased the integral myoelectric value of a 40% 1RM bench press by approximately 40%, similar to the value of an 80% 1RM bench press without BFR (Takarada et al., 2000). Furthermore, a study by Yasuda et al. showed an increase in the integral myoelectric value of the brachial triceps after a 30% 1RM bench press with pressure-restricted blood flow at arterial pressure, ranging from 40% to 60% of MVC (Yasuda et al., 2009). Although this study lacks assessments of CSA and integrated EMG values, the findings suggest that BFR-RST can improve measures of upper limb tensed arm circumference and BPP to a certain extent. Furthermore, since muscle strength growth is not solely dependent on increases in muscle volume but also on neural adaptations or the enhanced ability to recruit muscle fibers, it is plausible to hypothesize that improvements in BPP from BFR-RST may also be due to enhanced recruitment of type II muscle fibers. Additionally, the heart rate responses post-Wingate test indicate intragroup differences, reflecting the body’s recovery and fatigue resistance capabilities; these aspects will be further explored in conjunction with the anaerobic power section later.

### 4.2 Anaerobic power

The results of the Wingate test suggest that while both training protocols enhance upper limb anaerobic power, the improvements from the BFR protocol are more pronounced.

The enhancement of anaerobic power can be analyzed from the perspective of adaptive changes in type II muscle fibers. It is well established that type I and type II muscle fibers have different functional capacities, with type II fibers capable of significantly higher power output. Consequently, peak anaerobic power is associated with the proportion of type II muscle fibers (Robert et al., 1991). The acidic environment caused by metabolite accumulation during blood flow restriction and anaerobic conditions hinders the mobilization of type I fibers, leading to accelerated fatigue. Conversely, type II fibers can quickly replenish, maintaining muscle power by increasing their high-threshold activation. Additionally, the combined effects of ischemia and exercise mean that fatigue and subsequent low force output in some fibers lead to remaining force-producing fibers experiencing greater mechanical stresses and strains than what the nominally low load (30% of 1RM) would suggest (Scott et al., 2014). Thus, blood flow restriction training effectively recruits type II muscle fibers (Kang et al., 2015). Studies by Suga et al. and Yasuda et al. have shown that metabolic stress and hypoxia-ischemia in an ischemic-hypoxic environment lead to an increase in fast muscle fibers and cellular swelling, thus enhancing muscle hypertrophy (Suga et al., 2009). Yasuda et al. also found that BFR training increases the maximum independent activation rate of antagonist muscles, providing an optimal acidic environment for activating type II muscle fibers (Yasuda et al., 2009). A recent study by Copithorne et al. demonstrated that neuromuscular adaptations from muscle contractions resisted by low-intensity blood flow limitation were comparable to those from traditional high-load resistance exercise (Copithorne et al., 2012). This supports the finding that elevations in peak power (PP) and minimum power (MP) were greater under BFR in the EG group than in the CG group for the same RST training.

Additionally, the performance of anaerobic power also depends on the maximum rate of ATP (adenosine triphosphate) degradation and the maximum synthesis rate of CP (creatine phosphate) in the upper limbs. CP can convert to creatine under the catalysis of creatine kinase (CK), breaking down into a high-energy phosphate bond and releasing a phosphate group to supply energy for short-term high-intensity activity. Thus, Anaerobic power is also influenced by levels of CK. Notably, a recent study found that after five sets of blood flow-restricted exercise to volitional failure, the duration of torque decline, edema, and increase in blood proteins (CK and myoglobin) were prolonged (Sieljacks et al., 2016). Another study reported that a high-frequency training regimen of two sessions per day over five consecutive days led to significant increase in CK levels and a reduction in peak torque after 5 days (Bjørnsen et al., 2018). Notably, minimal changes were observed when these protocols were repeated 10–14 days later, suggesting a potential repeated bout effect with this training method. Therefore, these factors may partially explain why the anaerobic power improvements in the EG were more substantial than those in the CG in this study. Although case studies have reported that acute elevation of CK triggered by BFR could potentially lead to rhabdomyolysis (Clark and &Manini, 2016; Tabata et al., 2016), prevalence statistics from the literature suggest a very low occurrence probability (0.07%–0.2%) (Thompson et al., 2018). Furthermore, there is also no direct evidence indicating that BFR poses a higher injury risk than traditional exercise.

In the Wingate test, tPP typically appears 3–10 s after the start of the test and, along with the rate of anaerobic power decline (PD), serves as an indicator of fatigue resistance. After intense physical activity, half of the consumed phosphagen is resynthesized within 20–30 s and fully recovers within 3–4 min; thus, measuring heart rate post-test can reflect the body’s recovery ability following high-intensity anaerobic activity to some extent. The performance of PD is influenced by PP and MP, according to the formula: Rate of anaerobic power decline (%) = (Maximum anaerobic power − Minimum anaerobic power)/Maximum anaerobic power × 100%. A lower value indicates stronger fatigue resistance. Boxing matches typically consist of multiple high-intensity rounds, each lasting 2–3 min, during which athletes are required to perform frequent high-intensity punches and movements. A decrease in PD values ensures that boxers can maintain efficient punching even under fatigue. Moreover, lower PD values indicate that athletes are able to recover more quickly after high-intensity exercise, which is particularly crucial during the brief recovery periods (usually 1 minute) between rounds in a boxing match. Therefore, the calculation of PD values is not only of theoretical significance but also closely related to actual performance in boxing, especially regarding fatigue management and punching efficiency. In this study, significant changes in the indicators of tPP and PD (intergroup differences p < 0.05), and heart rate 30 s after the Wingate test (intragroup differences p < 0.05) all suggest that the EG has significantly enhanced fatigue resistance and recovery capabilities, linked to their increased PP and MP.

It is known that the local accumulation of metabolic byproducts, such as lactate and hydrogen ions, stimulates sympathetic nerve activity and the secretion of exercise-induced catecholamines (Cryer, 1991). The increase in lactate lowers the body’s pH, affecting the activity of enzymes such as phosphofructokinase, hexokinase, and pyruvate kinase (McArdle, 2006). This reduction in the glycolytic rate can lead to fatigue, manifested as a diminished ability to maintain original work capacity, resulting in elevated PD. Additionally, lactic acid can inhibit brain cell excitability and the transmission of excitation at nerve-muscle junctions, affecting muscle function. However, such effects are unlikely to occur rapidly (Maijiu, 2017). The results of this study reveal that after 4 weeks of intervention training, although both EG and CG showed increases in PP and MP, the fatigue resistance of EG was superior. This enhancement is related to adaptations in upper limb lactate or the enhanced ability to tolerate acidic environments induced by BFR. The improvements in the body’s anaerobic capacity, influenced by human adaptive mechanisms, also enhanced fatigue resistance (PD) and recovery ability (heart rate post-Wingate test). Research by Kazushige Goto et al., using a similar training method (10 repetitions of three to five sets of high-intensity exercise with 1 min of rest between sets), demonstrated a significant post-exercise increase in lactic acid, which decreased over time (Goto et al., 2005). This implies that the body’s lactic acid environment adapts physiologically after a period of training (approximately 4 weeks). However, this is speculative, and more accurate lactate testing experiments are needed for confirmation. These results suggest that 4 weeks of BFR-RST intervention training can indeed enhance upper limb fatigue resistance and the body’s recovery capabilities to a certain extent.

### 4.3 Punching performance

The results suggest that a 4-week repeated sprint training (RST) program may have a limited effect on peak punching speed, as peak speed is more reliant on neuromuscular coordination and technical proficiency rather than just strength or power improvements. Focusing on enhancing upper limb speed-strength or refining punching technique may yield better results in improving peak punching speed. Furthermore, These findings also indicate that both training methods can enhance the peak punching speed of boxers under fatigue conditions, but BFR-RST performs better.

It is established that phosphocreatine stores are largely depleted within 10 s of “all-out” exercise and only partially restored during recovery between sprints, a critical factor for manifestation of anaerobic capacity (Gaitanos et al., 1993). Research suggests that anaerobic glycolysis accounts for approximately 40% of the total ATP supply during the initial 6-s effort of a repeated sprint bout (Girard et al., 2011). Hence, the all-out punch at 6 s and the subsequent peak speed are primarily linked to anaerobic capacity. This concept is supported by a study by Sigitas Kamandulis et al., which found that 4 weeks of RST-specific training can enhance boxers’ repeated sprinting ability (Kamandulis et al., 2018). This enhancement is likely due to improvements in both aerobic and anaerobic capacities, as well as increased sprint efficiency. It was also observed that RST athletes did not exhibit lower lactate levels after simulated matches, indicating a potential increase in anaerobic capacity alongside improved aerobic capacity (Kamandulis et al., 2018). Although this study did not measure changes in aerobic capacity, the observed enhancement of anaerobic power supports their hypothesis.

Furthermore, the evidence that BFR training can enhance athletic performance is corroborated by other publications. Faiss et al. reported that 4 weeks of RSH (three sets of 5 × 10-s sprints) significantly increased the number of sets to exhaustion during repeated sprint tests, unlike training in normoxia (Faiss et al., 2013). Galvin et al. also found that 4 weeks of RSH yielded twice the improvement in distance during intermittent running tests compared to equivalent RSN (Galvin et al., 2013). Repeated sprint ability is linked to phosphocreatine (PCr) resynthesis and aerobic capacity (Mendez-Villanueva et al., 2012). During short-duration, high-intensity exercises lasting less than 60 s, the metabolic response under hypoxic conditions is proposed to diverge from that in normoxia, despite comparable exercise capacities (Weyand et al., 1999). Previous research indicates that power output during a 40-s maximal pedaling or a maximal anaerobic running test did not exhibit significant differences between hypoxic and normoxic conditions (Ogura et al., 2006; Ogawa et al., 2007). Nevertheless, the contribution of the anaerobic energy supply increased by approximately 9.3% in hypoxic conditions compared to normoxia. This enhanced anaerobic energy supply might provide a greater stimulus for adaptation and further improvement in repeated sprint ability. Moreover, recent discoveries indicate that performance in repeated sprinting exercises for athletes like elite soccer players, international rugby players, and cross-country skiers can be improved after only 2 weeks of an RSH (RST in ischemic hypoxic conditions) plan (Beard et al., 2019; Christensen et al., 2011; Faiss et al., 2015). Thus, the study results are in close alignment with these findings.

## 5 Practical applications

The results of this study indicate that both RST methods effectively enhance upper limb anaerobic power and strength in boxers, but the improvements from BFR are more pronounced than those from conventional RST. Moreover, neither training method significantly affects peak punching speed. However, in terms of fatigue resistance, the BFR-RST protocol is significantly more effective than conventional RST, due to the notable enhancement of anaerobic power.

## 6 Limitation

First of all, to reduce the potential bias caused by initial differences in physical fitness or training background, the independent sample T-test method was used in our study for grouping. Randomized controlled trial design can be implemented in future studies to further enhance the robustness of research results. Secondly, the selection of test indicators in this study is somewhat limited, only partially reflecting the upper limb performance of boxers. Future research could include measurements such as blood lactate concentration to assess fatigue resistance or incorporate 5 × 10-s sprint punches to evaluate repeated sprint ability and explore indicators such as muscle cross-sectional area to further assess the impact of BFR-RST on boxers’ performance. Finally, the experiment spanned only 4 weeks, and the effects of long-term training remain unclear, necessitating further studies to uncover the deeper mechanisms of long-term training effects.

## Data Availability

The original contributions presented in the study are included in the article/supplementary material, further inquiries can be directed to the corresponding authors.
